# The Relationship between Addiction and Socio-demographic Characteristics of Iranian Newcomer Prisoners

**DOI:** 10.5539/gjhs.v6n2p168

**Published:** 2013-12-24

**Authors:** Mehdi Amiri, Masoumeh Dejman, Mojgan Dastoury, Payam Roushanfekr

**Affiliations:** 1 Social Determinant of Health Research Center, University of Social Welfare and Rehabilitation Sciences, Tehran, Iran

**Keywords:** drug use, social deviances, sociology, prison

## Abstract

Prison has proven to be a suitable environment for the identification of various socio-demographic characteristics of those individuals whose drug use, or related crimes lead to incarceration. Furthermore, the prison environment could also support increased understanding of both the pattern and the relationship between drug use and the incidence of crime and deviances. Using the survey method, this study examines the socio-demographic features of 2200 prisoners in seven provinces of Iran. More specifically; drug abuse patterns and the relationship among addiction, crime prevalence, and some personal as well as socio-demographic characteristics were studied. According to the findings, characteristics such as age, education level, economic status, urban and/or rural status, all have an effects on the rate of drug use and, on crime commitment and its re-occurrence. Accordingly, younger age, lower socioeconomic status and urban residence showed a relationship with tendency to commit crime and repeat it while employment had no significant effect.

## 1. Introduction

There is little agreement among researchers on which social factors have the greatest impact on social problems, however, with regard to drug addiction, there is general agreement that considers a society which is experiencing addiction is vulnerable to a breakdown in power. As a result, this issue is so important that it has attracted the attention of different groups such as physicians, psychologists, sociologists, and police forces (Lietiery et al., 1980). Overall, there have been many studies conducted in this area, but there is still the need for further research, which should be addressed by; review updated information from related organizations, examine addiction from several different perspectives, apply research findings in the application of resource management, and develop efficient social and economic plans. Developing comprehensive plans to counteract drug addiction and drug trafficking requires paramount attention, particularly when research suggests that addiction is prevalent among youth and the fact that the addiction age has lowered considerably (Gravett, 1999). Using drugs is associated with social and ethical abnormalities; as a result, it endangers not only the health of the addict but it also affects larger society.

Therefore, each year, as the substance use increases in society, a lot of criminals are imprisoned in regard to drug abuse. Although two objectives of punishment and reform have been defined for prison (Abdi, 2002), the reality is that people enter a wide network of criminals after imprisonment, which can encourage them to commit more professional crimes according to differential association theory (Sutherland & Cressey, 1974). ‘This is because of inadequacy in the correctional and rehabilitating measures for prisoners, which turns prison into a school for learning crime. In fact, compared with when they first entered prison, prisoners are more prone to re-commit a crime’ (Ashouri, 2003). This issue gains greater importance when one considers that, ‘both the absolute and the relative number of prisoners are increasing in many societies’ (Gassin, Cimamonti, & Bonfils, 2011). Considering the financial aspects, such growth is devastating and has widespread destructive results. This becomes even more serious if the prison environment intensifies the tendency to commit a crime. Under such circumstances, we are moving against the social goals of society which requires us to take preventive measures.

One of the most effective measures in this regard is to gain a broader view of the status quo and to explore factors intensifying drug use related crimes in society. Prison is utilized as the main form of punishment throughout the world (Abdi, 2002) and they are expected to hold a great number of individuals who have addiction related convictions (Birchard, 2001; Reindollar, 1999; Birchard, 2003; Madani, 2008). As a result, prison may serve as one of the most suitable places for identifying socio-demographic and socio-cultural characteristics of prisoners linked to drug use and drug related crimes, as well as the pattern and the relationship among these factors and the type and incidence of crime and deviance. Relevant studies can help policy-makers and executives, by informing them of the socio-demographic characteristics of those prisoners that have addictions and addiction/drug related crimes, thus identifying groups who are more likely to experience drug addiction. They can also take crucial steps to both prevent and reduce the harm and build a secure and healthy society. Thus, in the present study, the relationship between drug use and the socio-demographic characteristics of drug users entering different prisons in 7 major prisons in Iran January in 2009 were examined.

### 1.1 Theoretical Framework

The relationship between crime commitment and drug misuse has been studied and supported in many studies. Three models of drug-related crime have been suggested; psychopharmacologic, systemic, and economically compulsive. The economically compulsive model of violence holds that some drug users engage in economically oriented violent crime to support their drug use. This model has been demonstrated in a number of studies where drug use and sales, property crimes and prostitution are the primary economic offenses committed by users, however, armed robberies and muggings may also occur (Inciardi, 2001)

According to the Drug Enslavement Theory, drug users have no other choice than to enter the world of crime since they cannot afford the prohibitive cost of obtaining drugs through legal ways. The latter seems more prevalent among poor drug addicts who not only lack enough educational and professional skills, but also suffer from social discrimination and other social deprivation. This stands in contrast to those who use drugs just for pleasure (Sedigh Sarvestani, 2011). The General Deviance Syndrome Theory also shows a high correlation between drug use and crime commitment due to a person’s tendency for deviation. Thus, drug use by itself is not a reason for crime commitment, but it does reinforce the tendency to commit it (Goode, 2012).

Researchers of juvenile delinquency studies believe that drug use and vandalism are the most prevalent deviant behaviors among youth (Santrok, 2011; Siegel & Senna., 2011). Using drugs for pleasure or to help them adjust to difficult psychological and social conditions, many youths have undergone serious losses in their lives. Young people have a burning desire for independence, which is at odds with their extended period of education and lack of employment. Moreover, parental control supports a delay in a young person’s sense of responsibility. All these factors can influence the use of drugs in youths (Gonet, 1994). An extensive research study on the life cycle of drug addiction indicated that the beginnings of addiction occur in late teenage and early adulthood years since the person is under a great deal of pressure from many difference sources such as role strains from deciding upon; sexual relationships, adulthood responsibilities, social relationships, family/school/work conditions, as well as the pressure resulting from role deprivation and a lack of recognized behavioral patterns (Winick, 2013). According to sociologists, the increasing use of drugs is rooted in forces that are effective in the decline of legitimate values, conventional norms, and social controlling bodies, as well as in structural forces that provide opportunities for youth to have greater freedom away from the usual controlling mechanisms. In this sense, drug use and related guidelines indicate a social change process (Lietiery et al., 1980).

On the other hand, the physical environment and social development of urban areas leads to social and cultural heterogeneity, which has a considerable influence on producing urban deviance. The type of accommodation, their surrounding social milieu, poorly paid work social failures, and economic poverty, paves the way for social harm and deviance such as drug addiction (Nassiri, 2003).

Shaw and Mckey (Shoemaker, 1996), used an ecological point of view when considering deviant behavior of human groups based on their urban environment in megalopolises. Ahmadi (2010) believes that the sprawl of megalopolises, the tendency of migrating populations to inhabit such cities, and unsuitable accommodation conditions in metropolitan fringes, have developed into particularly unhealthy places, and in turn, this increases the prevalence of delinquency and crime in all parts of the city including the center and its surroundings, and this has been the case in many megalopolises in developing countries. Dramatic changes in cities, which are mostly the consequences of industrialization, have turned megalopolises into great cultural centers for change (Sotoudeh, 2011); furthermore, industrialization and technical development has exceeded the speed of cultural development. Such cultural lags, resulting in deviance and social disruption including vagrancy, vandalism, drug addiction, and drug trafficking (Tavassoli, 2006) results in urban etiquette not being internalized among its citizens or embodied in their behaviors and attitudes.

## 2. Methodology

The present descriptive study was carried out as part of a comprehensive study aimed at examining the effects of an intervention trial for harm reduction on all new prisoners from seven prisons in seven Iranian provinces (n=2200) in January 2009. It was conducted in two stages using before-after method in two months.

These prisons were located in Karaj, Gorgan, Zahedan, Urmia, Bandar Abbas, Yazd, and Kermanshah. In the first stage, all new inmates in these 7 prisons (totally 2200 people) were studied (Table 1). This paper is the result of the first stage of the study.

**Table 1:** Frequency distribution of the study samples in seven provinces in Iran in 2009

Prison	Zahedan	Urumiyeh	Bandar Abbas	Yazd	Gorgan	Karaj	Kermanshah	Total
Number of participants in the sample	200	200	300	300	400	500	300	2200

The main technique in this study was interview using questionnaires by trained in-prison health officials who interviewed newly admitted prisoners to the 7 prisons. Having obtained the prisoners’ consent to participate in the study, drug tests using 4-medication kits were conducted to complement the interviews. Each prisoner and prison facility was given an identification number by trained personnel of the Health and Hygiene Division in their respective prisons. In the first phase, the tool was a questionnaire and its validity was approved by officials and experts in the prisons studied. An Addiction Diagnostic Test was conducted on the participants using four-medicine kits (Note 1) for all new inmates in these 7 prisons (n=2200). To avoid discrepancies between kits manufactured by different companies, 3,500 ACON brand kits manufactured by Azma Kosar Company were used.

All questionnaire data were analyzed by SPSS software (Version 16). Furthermore, descriptive analyses and correlation among the variables were also calculated (Chi-square test, mean, frequency).

### 2.1 Variable Defenition:

Crime: Legally, crime is any action or inaction that deserves punishment according to law (Article 2 Islamic Punishment Code, July 30th, 1991)

Prison: Based on article 3 of Regulations of Prisons and Security and Redemption Measures, December 11th, 2005, is a place where people with definite convictions are taken to by the order of authorities in judiciary for a certain time or permanently in order to do their term, learn skills, rehabilitate and re-adapt.

## 3. Findings

According to the findings of the present study, only 6.3% of the prisoners (n=139) were arrested for drug addiction. However, the Addiction Diagnostic Test with four-medicine kits revealed that 65.8% of the prisoners (n=1440) had a record of drug use, which was 13.6% more frequent among urban prisoners (n=1209, 68.5% of the drug user population) than rural prisoners (n=231, 54.9% of the drug user population). It was also evident that 56.7% of the studied prisoners (n=1248) were users of substances such as morphine, cannabis, and/or amphetamine. Morphine, the most popular drug, was used by 52.4% of the prisoners (n=1153); followed by cannabis by 9.2% (n=203), and, finally, amphetamine by 5.3% (n=117). The results of the fourth kit (benzodiazepine) were positive in 23.4% of the cases. The use of such drugs does not necessarily indicate that the person is a user of narcotics, so the related figures have been omitted. However, this pattern was different from that of the studied population after two months of imprisonment where the pattern changed to 38.7% for heroin use, 27.4% for opium use, and approximately 40% for cannabis and amphetamine use.

A total of 99% of the respondents were male and with regard to the prisoners’ age, this study showed that, out of 1443 participants who entered prison in the year 2009, 7.2% were younger than 20 years and a further 87.3% were younger than 40 years (X^2^=11.208, df=3, p=0.011). The results also demonstrated that drug use rates closely co-varies with that of crimes in different age groups, and 65.8% of newcomer prisoners were drug users (Table 2).

**Table 2:** Frequency distribution of crime and prevalence of drug addiction rates based on age in 7 Iranian prisons in 2009

Age	Younger than 20	20-30	30-40	Older than 40	Total
Number of addicts	104 (7.2%)	745 (51.6%)	411 (28.5%)	183 (12.7%)	1443 (65.8%)
Total number of crimes	173 (7.9%)	1106 50.4%)	605 (27.6%)	310 (14.1%)	2194 (100%) (Note 2)

The present study showed that, out of 1440 drug users in these prisons in the year 2009, 84% (n=1 209) were from urban areas whereas only 16% (n=231) came from rural areas. This figure is 15.5% higher than the National Statistics of urban populations of 68.5% according to Population and Housing Census 2006. Moreover, among all 985 prisoners with a past record of imprisonment, the population of urban prisoners with the rate of 83.4% (n=821) was 14.9% more than the National Statistics (68.5%). As a result, it can be asserted that in the current studied population, an urban status had a positive relationship with both drug use and crime re-occurrence.

**Table 3:** Frequency of drug users based on location and the prison records in newcomer prisoners in 7 Provinces of Iran in 2009 and its comparison with national statistics

Location	National Statistics (percentage)	Drug addicts in prisons (percentage)	Difference with National Statistics	Prisoners with prison records (percentage)	Difference with National Statistics
Urban population	68.5%	84% (1209)	15.5%	83.4% (821)	14.9%
Rural population	31.5%	16% (231)	15.5%	16.6% (164)	14.9%
Total	100%	1 440 (100%)	-	985 (100%)	-

The findings of the present study also maintain that there is a relationship between higher levels of education, and lower numbers of drug users. Out of the 1442 participants in this study, 82% (n=1180) had an educational degree less than a diploma, while 18% (n=262) had a diploma degree or higher. Hence, it can be concluded that there was an inverse relationship between education levels and the rate of drug use in the studied population (X^2^=44.100, df=6, p=0.000). Furthermore, the present research showed that 40.5% of the participants had their own private house/apartment while 59.5% were either living in rented houses/apartments, residing with their relatives, or were without a residence/homeless (X^2^=44.100, df=6, p=0.000). The average income of the participants was 3 790 000 Rials (about US $396).

Finally, the findings suggest that there was no significant relationship in regard to prisoners’ employment status, either employed or unemployed (X^2^=17.766, df=2, p=0.000).

**Table 4:** Frequency distribution of drug use record based on job status among newcomer prisoners in 7 large Iranian provinces in 2009

Job status	Prisoners with drug use records (percentage)	Prisoners without drug use records (percentage)	Total
Employed	65.8% (1149)	34.2% (596)	100% (1745)
Unemployed due to military service	44.7% (34)	55.3% (42)	100% (77)
Unemployed	69.9% (258)	30.1% (111)	100% (369)
Total	65.8% (1441)	34.2% (749)	100% (2190) (Note 3)

## 4. Discussion

The findings of the present study which was carried out on 2 200 prisoners from seven large prisons in Iran in January 2009, indicated that although 6.3% of the studied population were jailed for drug addiction, the drug consumption test for most prisoners was positive, and approximately 65.8% of prisoners used drugs such as morphine, cannabis, and amphetamine (amphetamine had the highest popularity). The amount of benzodiazepine use (23.4%) among the samples showed that about one-fourth of newcomer prisoners had used this drug for neurological and psychological disorders and diseases like hypnological disorders, paroxysm, stress, and/or anxiety. However, this pattern changed after two months; heroin, opium, cannabis, and crystal, were used respectively, and had become the most frequently used drugs among prisoners. It is apparent that drug availability patterns for prisoners were different in and out of prison. The results demonstrated that drug use closely co-varies with that of crimes in different age groups. In other words, the number of newcomer prisoners and drug users in different age groups showed little difference. In addition, the rate of drug use with a past record of imprisonment were 15.5% and 14.9%, respectively, higher in the urban population in comparison with the rural population, which indicates a positive relationship between urban status and drug use as well as crime re-occurrence. The findings of the present research did not show a significant difference between the number of the employed and unemployed prisoners among the population of drug users. Hence, unemployment could not be regarded as an influential variable on using drugs in the studied population. However, this was not the case for other influential variables affecting economic and social status; in other words, there was an inverse relationship between the level of education and using drugs: the rate of using drugs was 4.6 times higher among those who had a degree below diploma level than in those with a diploma or a higher degree. Moreover, about 60% of the studied population did not own a private house/apartment and the average income of addicts was 3 790000 Rials (about US $396), based on US dollars, 9 570 Rials (official price). The monthly income of 75% of the prisoners was less than 4 000 000 Rials (about US $418).

From a sociological perspective and according to the structural-functional analysis, there is a close relationship between deviances’ type, pattern, and dispersion with the features of socio-economic and socio-demographic organizations. Researches findings (Sadat Ghoreishi, Ahmadvand, & Sepehrmanesh, 2010; Nakha’ee, 2002; Shaw & Mckey as cited in Shoemaker, 1996; Kaffashi & Eslami, 2009; Hemmasi & Kessler, 1997) in most societies, including Iran, have shown that patterns of deviant and criminal behaviors vary based on the socio-economic conditions of the areas.

From such a point of view, an extensive research on the life cycle of drug addiction indicated that the experience of using drugs has an apparently constant relationship with the age variable (Braucht et al., 1995; Santrok, 2001; Siegel & Senna, 2011; Gonnet, 1994), and the beginning of addiction is in late teenage and early adulthood years (Winick, 2013). Age ranges of these prisoners who were from seven provinces of Iran were also examined. The results showed that most prisoners (87%) were below the age of 40 years, indicating that as age increases, the rate of drug use decreases (Table 2).

In the report of the research scheme against drug addiction (Mehriar & Jazayeri, 2007), it was maintained that drug use was the underlying reason for many crimes such as murder, larceny and rape. There is a large body of research from all over the world which supports the correlation between addiction and crime commitment (Meijer et al., 2003; Harrison, 1992; Wren, 1999). In this study, the drug consumption test for most participants (65.8%) was positive; in addition, the close relationship of drug use with the rate of crime among newcomer prisoners in different age groups (Table 2) supports the same idea.

On the other hand, the physical and social development of the cities and population centers leads to social and cultural heterogeneity, which has considerable influence on producing urban crime and deviance. Findings of sociological studies (Nassiri, 2003; Shaw and Mckey as cited in Shoemaker, 1996; Ahmadi, 2010; Tavassoli, 2006) indicate that the sprawl of migration to megalopolises, dramatic changes of industrialization and cultural lag paves the way for social harm and deviances such as drug use. According to the findings of the present study, multi-faceted effects of cities’ development and spread of deviances are best demonstrated in the significant difference between the ratios of urban newcomer prisoners to those of rural areas as well as urban prisoners’ crime commitment records that were 14.9% higher than the urban population. Supporting previous studies, the current study indicated that urban population, in comparison with rural population, were more prone to undergo drug use and experience re-occurrence of crimes and deviances.

The findings of this study also demonstrated that the majority of the participants had a low level of education, did not own a private house/apartment, and their average income was 3 790 000 Rials (about US $396). The poverty line in 2007-2009 was estimated to be 3870000 Rials based on income distribution and family consumption (Mohammadzadeh, Fallahi, & Hekmat, 2011). However, this figure, for the absolute poverty line, is an average figure for the whole country, but for Tehran and other megalopolises, this figure might increase up to 20% for housing and commuting expenses. Based on the aforementioned data, it could be concluded that the income of a great number of the population under study was below the poverty line.

The results of previous research have revealed that drug use has a strong relationship with experiencing social deprivations and being in a marginalized economic status (Currie, 1993). As the results of this present study indicate, a considerable number of addicts included in this study were unemployed and had low educational levels. These people live under difficult conditions which include poverty and social deprivation, as a consequence of their addiction this led them to commit crimes, and they were imprisoned as a result.

## 5. Conclusion

In sum, the results of this study on 2200 new inmates of 7 major prisons in Iran shows that demographic characteristics are closely related to drug use and committing crime. Therefore, the youth, as the most important human and social asset of the country, are the most vulnerable and have greater tendency to use drugs and commit crime. That is why it is essential that authorities control the increasing trend of this tendency in order to achieve a healthier society. The higher number of inmates that use drugs among city residents and people with low material and cultural assets shows that it is necessary to control the problems caused by expansion of cities and prioritize the programs to develop the community economically, socially and culturally to prevent expansion of social deviations.

## 6. Recommendations:

According to the findings obtained by this study, and results from studies conducted in Iran as well as in other countries, the following suggestions are aimed at preventing or decreasing social problems and creating a healthier society:

Prevention strategies should be given priority over treatment/cure. Prevention measures are less expensive and entail fewer resources, whereas treatment/curing and rehabilitation programs require different specialties and are expensive. Moreover, such programs only cover relatively few numbers of people, and outcomes are less effective.

Removing poverty from villages, and establishing vocational and educational facilities for rural residents would avoid large-scale migration to the cities and the consequences of unbalanced growth.

In order to elevate cultural capital of the society, it is recommended that cultural, ethical and religious development programs be conducted for families and individuals through the development of educational and cultural facilities

## Footnotes

Note 1. These kits rapidly showed if a person had used a main narcotic substance including: opium, morphine, cannabis or amphetamine during the last 72 hours. Each kit showed exclusively a certain narcotic substance in the user.
